# Draft Genome Sequences of 40 *Pseudomonas aeruginosa* Clinical Strains Isolated from the Sputum of a Single Cystic Fibrosis Patient Over an 8-Year Period

**DOI:** 10.1128/genomeA.01205-16

**Published:** 2016-12-15

**Authors:** Irene Bianconi, Silvia D’Arcangelo, Mattia Benedet, Kate E. Bailey, Alfonso Esposito, Elena Piffer, Alex Mariotto, Ermanno Baldo, Grazia Dinnella, Paola Gualdi, Michele Schinella, Claudio Donati, Olivier Jousson

**Affiliations:** a Centre for Integrative Biology (CIBIO), University of Trento, Trento, Italy; b Trentino Cystic Fibrosis Support Centre, Rovereto Hospital, Trento, Italy; c Operative Unit of Clinical Pathology, Rovereto Hospital, Trento, Italy; d Fondazione Edmund Mach, San Michele all’Adige, Italy

## Abstract

We report draft genome sequences of 40 *Pseudomonas aeruginosa* strains, isolated from the sputum of a single cystic fibrosis patient over eight years. Analyses indicated a correlation between multidrug-resistant phenotypes and population structure. Our data provide new insights into the mechanisms leading to acquisition of antibiotic resistance in *P. aeruginosa*.

## GENOME ANNOUNCEMENT

*Pseudomonas aeruginosa* is the most pervasive of all recognized pathogens in the nosocomial environment, causing pulmonary and bloodstream infection with mortality rates of up to 50% (1). Multi-drug-resistant (MDR) *P. aeruginosa* strains are emerging with increasing frequency and infection rates have tripled over the past two decades (2, 3). Some *P. aeruginosa* strains have been found to be resistant to nearly all or all antibiotics in clinical use (4).

Cystic fibrosis (CF) patients infected with resistant *P. aeruginosa* are exposed to increased mortality and morbidity (5, 6) and estimates indicate that 25 to 45% of adult CF patients are chronically infected with MDR *P. aeruginosa* within their airway (7). The bacterium develops MDR phenotypes during its persistence in a CF patient’s airway by accumulating pathoadaptive mutations (8). Whole-genome sequencing (WGS) can help to point out potential molecular mechanisms of resistance and has already proved to be able to predict antimicrobial susceptibility in several pathogens (9, 10). However, despite the fact that several WGS studies on *P. aeruginosa* CF lineages have been published (11–14), their evolutionary trajectories in relation to the development of antimicrobial resistance remain mostly unexplored to date.

To track the pathoadaptive changes leading to the development of MDR in *P. aeruginosa* during its microevolution in a CF patient’s airway, we obtained whole-genome sequences of 40 *P. aeruginosa* clinical CF strains isolated at Trentino Regional Support CF Centre (Rovereto, Italy) from the sputum of a single CF patient over an eight-year period (2007 to 2014). Interestingly, despite a high degree of genome sequence conservation, isolates evolved toward the acquisition of an MDR phenotype over time.

Bacteria were grown in Luria-Bertani broth overnight at 37°C in a shaking incubator. Cells were harvested and genomic DNA was extracted using the DNeasy blood and tissue kit (Qiagen, Germany) following the manufacturer’s instructions for Gram-negative bacteria. Genomic DNA libraries were prepared using the Nextera XT DNA library preparation kit and protocols (Illumina, USA) and sequenced on the Illumina MiSeq platform at the Next Generation Sequencing (NGS) Core Facility of the Centre for Integrative Biology, University of Trento. Assembly of draft genomes was carried out using SPAdes version 3.1.0 (15). To improve the assemblies’ qualities, raw reads were mapped on the contigs using Bowtie2 v2.2.6 (16) and contigs with less than three reads mapping and/or with coverage below 1 were removed.

Identification of MLST profiles (sequence types) was performed *in silico* from *de novo* assembled genomes using MLST 1.8 (Table 1) (17).

**TABLE 1:** Draft genome sequences and global statistics of the 40 *P. aeruginosa* CF isolates

Accession no.	Isolate name	Yr of isolation	Sequence type	No. of contigs	Genome size (kb)	*N*_50_ (kb)	G+C content (%)
MAUO00000000	TNCF_3	2007	390	139	6,636	92	66.28
MAUP00000000	TNCF_4M	2007	390	161	6,630	78	66.29
MAUQ00000000	TNCF_6	2007	390	356	6,618	31	66.28
MAUR00000000	TNCF_7M	2007	390	259	6,623	47	66.28
MAUS00000000	TNCF_10	2007	390	101	6,643	143	66.28
MAUT00000000	TNCF_10M	2007	390	107	6,633	111	66.29
MAZG00000000	TNCF_12	2007	390	102	6,545	177	66.36
MAZI00000000	TNCF_13	2007	390	75	6,637	195	66.27
MAZH00000000	TNCF_14	2007	390	89	6,633	158	66.28
MAKL00000000	TNCF_16	2007	1864	59	6,638	269	66.28
MAZJ00000000	TNCF_23	2007	390	71	6,635	228	66.28
MAZK00000000	TNCF_23M	2007	390	64	6,636	228	66.28
MAKM00000000	TNCF_32	2007	390	67	6,639	229	66.28
MAZL00000000	TNCF_32M	2007	390	138	6,627	93	66.28
MAZM00000000	TNCF_42	2008	390	70	6,639	228	66.28
MAZN00000000	TNCF_42M	2008	390	71	6,640	228	66.28
MAZO00000000	TNCF_49M	2008	390	76	6,635	177	66.29
MAZP00000000	TNCF_68	2010	390	82	6,633	162	66.28
MAZQ00000000	TNCF_69	2010	1863	88	6,639	150	66.28
MAZR00000000	TNCF_76	2010	390	61	6,634	281	66.28
MAZS00000000	TNCF_85	2010	1864	101	6,644	124	66.29
MAZT00000000	TNCF_88M	2010	1864	65	6,636	229	66.28
MAZU00000000	TNCF_101	2011	1864	142	6,653	92	66.28
MAZV00000000	TNCF_105	2011	390	92	6,644	191	66.28
MAZW00000000	TNCF_106	2011	390	77	6,634	205	66.28
MAZX00000000	TNCF_109	2011	390	69	6,634	205	66.28
MAZD00000000	TNCF_130	2012	390	157	6,625	76	66.28
MAZF00000000	TNCF_133	2012	390	82	6,637	154	66.29
MAZE00000000	TNCF_133_1	2012	1864	87	6,641	269	66.28
MAKK00000000	TNCF_151	2013	390	53	6,629	378	66.28
MBMI00000000	TNCF_151M	2013	1864	103	6,636	143	66.28
MBMJ00000000	TNCF_154	2013	390	86	6,635	177	66.28
MBMK00000000	TNCF_155	2013	390	62	6,634	339	66.28
MBML00000000	TNCF_155_1	2013	1923	71	6,635	221	66.28
MBMM00000000	TNCF_165	2013	1923	119	6,634	135	66.28
MBMN00000000	TNCF_167	2013	390	73	6,634	191	66.27
MBMO00000000	TNCF_167_1	2013	390	91	6,628	143	66.28
MBMP00000000	TNCF_174	2014	390	111	6,645	143	66.29
MBMQ00000000	TNCF_175	2014	390	118	6,642	124	66.28
MBMR00000000	TNCF_176	2014	1923	61	6,637	354	66.28

The average number of contigs per genome was 101 with a standard deviation of 56. Draft genomes ranged in size from 6,545 kbp to 6,653 kb with a G+C content of 66.28% (Table 1). The *N*_50_ of the draft genomes ranged from 30,645 to 378,317 bp with an average of 179.843 bp (Table 1).

### Accession number(s).

This whole-genome shotgun project has been deposited at DDBJ/ENA/GenBank. See Table 1 for accession numbers of the single genomes. The version described in this paper is the first one.

## References

[1] Osmon S, Ward S, Fraser VJ, Kollef MH 2004 Hospital mortality for patients with bacteremia due to *Staphylococcus aureus* or *Pseudomonas aeruginosa*. Chest 125:607–616. doi:10.1378/chest.125.2.607. 14769745

[2] Obritsch MD, Fish DN, MacLaren R, Jung R 2005 Nosocomial infections due to multidrug-resistant *Pseudomonas aeruginosa*: epidemiology and treatment options. Pharmacotherapy 25:1353–1364. doi:10.1592/phco.2005.25.10.1353. 16185180

[3] Lautenbach E, Weiner MG, Nachamkin I, Bilker WB, Sheridan A, Fishman NO 2006 Imipenem resistance among *Pseudomonas aeruginosa* isolates: risk factors for infection and impact of resistance on clinical and economic outcomes. Infect Control Hosp Epidemiol 27:893–900. doi:10.1086/507274. 16941312

[4] Centers for Disease Control and Prevention 2013 Antibiotic resistance threats in the United States, 2013. CDC, Atlanta, GA.

[5] Lyczak JB, Cannon CL, Pier GB 2000 Establishment of *Pseudomonas aeruginosa* infection: lessons from a versatile opportunist. Microbes Infect 2:1051–1060. doi:10.1016/S1286-4579(00)01259-4. 10967285

[6] Chmiel JF, Davis PB 2003 State of the art: why do the lungs of patients with cystic fibrosis become infected and why can’t they clear the infection? Respir Res 4:8. doi:10.1186/1465-9921-4-8. 14511398 PMC203156

[7] Lechtzin N, John M, Irizarry R, Merlo C, Diette GB, Boyle MP 2006 Outcomes of adults with cystic fibrosis infected with antibiotic-resistant *Pseudomonas aeruginosa*. Respiration 73:27–33. doi:10.1159/000087686. 16113513

[8] Breidenstein EBM, de la Fuente-Núñez C, Hancock RE 2011 *Pseudomonas aeruginosa*: all roads lead to resistance. Trends Microbiol 19:419–426. doi:10.1016/j.tim.2011.04.005. 21664819

[9] Stoesser N, Batty EM, Eyre DW, Morgan M, Wyllie DH, Del Ojo Elias C, Johnson JR, Walker AS, Peto TEA, Crook DW 2013 Predicting antimicrobial susceptibilities for *Escherichia coli* and *Klebsiella pneumoniae* isolates using whole genomic sequence data. J Antimicrob Chemother 68:2234–2244. doi:10.1093/jac/dkt180. 23722448 PMC3772739

[10] Zankari E, Hasman H, Kaas RS, Seyfarth AM, Agersø Y, Lund O, Larsen MV, Aarestrup FM 2013 Genotyping using whole-genome sequencing is a realistic alternative to surveillance based on phenotypic antimicrobial susceptibility testing. J Antimicrob Chemother 68:771–777. doi:10.1093/jac/dks496. 23233485

[11] Darch SE, McNally A, Harrison F, Corander J, Barr HL, Paszkiewicz K, Holden S, Fogarty A, Crusz SA, Diggle SP 2015 Recombination is a key driver of genomic and phenotypic diversity in a *Pseudomonas aeruginosa* population during cystic fibrosis infection. Sci Rep 5:7649. doi:10.1038/srep07649. 25578031 PMC4289893

[12] Marvig RL, Sommer LM, Molin S, Johansen HK 2015 Convergent evolution and adaptation of *Pseudomonas aeruginosa* within patients with cystic fibrosis. Nat Genet 47:57–64. doi:10.1038/ng.3148. 25401299

[13] Williams D, Evans B, Haldenby S, Walshaw MJ, Brockhurst MA, Winstanley C, Paterson S 2015 Divergent, coexisting *Pseudomonas aeruginosa* lineages in chronic cystic fibrosis lung infections. Am J Respir Crit Care Med 191:775–785. doi:10.1164/rccm.201409-1646OC. 25590983 PMC4407486

[14] Caballero JD, Clark ST, Coburn B, Zhang Y, Wang PW, Donaldson SL, Elizabeth Tullis D, Yau YCW, Waters VJ, Hwang DM, Guttman DS 2015 Selective sweeps and parallel pathoadaptation drive pseudomonas aeruginosa evolution in the cystic fibrosis lung. mBio 6:e00981-15. 26330513 10.1128/mBio.00981-15 PMC4556809

[15] Bankevich A, Nurk S, Antipov D, Gurevich AA, Dvorkin M, Kulikov AS, Lesin VM, Nikolenko SI, Pham S, Prjibelski AD, Pyshkin AV, Sirotkin AV, Vyahhi N, Tesler G, Alekseyev MA, Pevzner PA 2012 SPAdes: a new genome assembly algorithm and its applications to single-cell sequencing. J Comput Biol 19:455–477. doi:10.1089/cmb.2012.0021. 22506599 PMC3342519

[16] Langmead B, Salzberg SL 2012 Fast gapped-read alignment with Bowtie 2. Nat Methods 9:357–359. doi:10.1038/nmeth.1923. 22388286 PMC3322381

[17] Larsen MV, Cosentino S, Rasmussen S, Friis C, Hasman H, Marvig RL, Jelsbak L, Sicheritz-Pontén T, Ussery DW, Aarestrup FM, Lund O 2012 Multilocus sequence typing of total-genome-sequenced bacteria. J Clin Microbiol 50:135–1361. 10.1128/JCM.06094-11 PMC3318499 22238442

